# People Evaluate Agents Based on the Algorithms That Drive Their Behavior

**DOI:** 10.1162/opmi.a.26

**Published:** 2025-08-29

**Authors:** Eric Bigelow, Tomer Ullman

**Affiliations:** Department of Psychology, Harvard University, Cambridge, MA, USA

**Keywords:** Turing Test, generalization, program learning, theory of mind, intelligence attribution

## Abstract

When people see an agent perform a task, do they care if the underlying algorithm driving it is ‘intelligent’ or not? More generally, when people intuitively evaluate the performance of others, do they value external performance metrics (intuitive behaviorism) or do they also take into account the underlying algorithm driving the agent’s behavior (intuitive cognitivism)? We propose 3 dimensions for examining this distinction: Action Efficiency, Representation Efficiency, and Generalization. Across 3 tasks (*N* = 598), we showed people pairs of maze-solving agents, together with the programs driving the agents’ behavior. Participants were asked to pick the ‘better’ of the two programs, based on a single example of the two programs, evaluated on the same maze. Each pair of programs varied along one of our 3 proposed dimensions. Our framework predicts people’s choice of program across the tasks, and the results support the idea that people are intuitive cognitivists.

## INTRODUCTION

Suppose you’re visiting the lab of a top-notch engineer to witness a demonstration of two sandwich-making robots they’ve been working on. The engineer turns the robots on, and you see them making a sandwich in the *exact* same way. When the demonstration is concluded, the engineer turns to you and asks: ‘Which robot do you think is better?’ The question seems unanswerable. Or, the answer is simply ‘neither’ or ‘both’. But now, suppose the engineer gave you a chance to peek at the code that is driving the behavior of the robots. You see that one robot has an extremely long list of instructions, specific to the particular set-up of the current table, materials, and sandwich. The other robot, by contrast, has a shorter list of instructions that could solve a range of problems, including the behavior you just saw. Would seeing the programs that drive the robots affect your decision of which robot is better? And if so, which features of the program matter, specifically?

**Figure 1.**
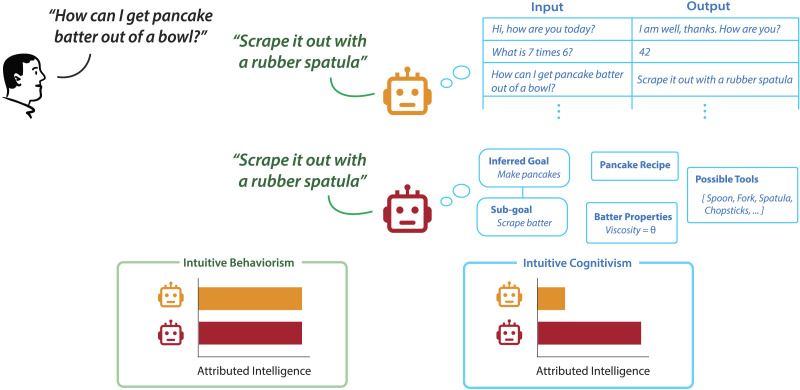
**People’s evaluation of agent behavior.** Consider two agents that produce the same outward behavior (depicted here as 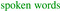), but with different internal programs or thought processes (depicted here as 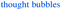). The 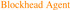 relies on a memorized lookup table of responses, whereas the 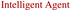 follows more sophisticated cognitive algorithms to generate its responses. A person who values only the performance of the agent (‘Intuitive Behaviorism’) would evaluate both agents as the same, whereas a person who also values the programs that underlie behavior (‘Intuitive Cognitivism’) would not see them as equal.

In this work, we examine whether people intuitively evaluate the behavior of agents based on external performance alone, or whether they also take into account the program that is driving that behavior, and if the latter, which features of the program matter. To put it in terms of the major research programs that guided psychology in the 20th century, our question is whether people are ‘Intuitive Behaviorists’ or ‘Intuitive Cognitivists’ (Figure 1). Our main hypothesis is that people do care about the algorithms or programs that drive behavior, above and beyond behavioral performance, and we study in particular what features of a program matter to people. We tested people’s evaluation of agent behaviors and the programs that drove those behaviors by having people observe the behavior of artificial agents solving maze tasks, along with the programs that generated the agent behaviors. We examined people’s sensitivity to external performance, as well as two key features of the programs. The first feature was equivalent to program length, in line with many previous proposals in cognitive science. The second feature was program generalization, which measures how many other problems the agent could solve beyond the specific one it is facing.

The structure of this paper is as follows. First, we describe past work related to the evaluation of agents in cognitive science. We then survey related past work on the evaluation of agents in artificial intelligence and philosophy, including the Turing Test and the Blockhead objection to the Turing Test. We consider the parallels between the Blockhead objection and contemporary research on the evaluation of agents in cognitive science and AI. With this background in hand, we formally describe three metrics that we propose people use to evaluate programs: Action Efficiency, Representation Efficiency, and Generalization. We report the results of two behavioral experiments that examined our three metrics using a novel set-up, featuring block programs and maze-solving agents as stimuli. We conclude by discussing the relationship between program length and generalization, as well as the significance of our work to cognitive science and current research on machine intelligence.

## BACKGROUND

### Evaluating the Actions of Agents in Cognitive Science

While our opening example involved robots, to build intuition, our main question is not actually about robots specifically, but rather how people intuitively evaluate the behavior of any agent. Recent work in cognitive science studying social reasoning posits that people understand many of the actions of others as being driven by structured plans, made up of beliefs, goals, and intentions (e.g., Baker et al., 2009, 2017; Jara-Ettinger et al., 2020; Kryven et al., 2021). Other work has suggested that people are also sensitive to cases where others may be using scripts or habits that are more similar to look-up tables than full model-based planning (e.g., Bass et al., 2024; Gershman et al., 2016; Schank & Abelson, 2013; Ullman & Bass, 2024). Such frameworks have quantitatively and qualitatively explained many behavioral patterns observed in how people reason about agents, but have not specifically focused on whether and how people value the internal workings of the planning programs that guide behavior. Here, we aim to test this question more directly by exposing participants directly to both the observed actions of an agent and the particular operations that caused the agent’s behavior. We note that there is a branch of related work in computational cognitive science, which considers concepts as programs, and assumes that people are performing a kind of program induction when reasoning and learning (Lake et al., 2017; Tenenbaum et al., 2011; Ullman & Tenenbaum, 2020). Bayesian program learning models are used to explain human behavioral performance, but typically the specific programming language is obscured to human participants, and is assumed to be a ‘language of thought’ (Quilty-Dunn et al., 2023) that people probably do not have direct access to. Here, we explicitly teach participants a simple programming language that controls agent behaviors. This enables us to test what features of programs, if any, people value when assessing agents.

There have been several recent works in computational cognitive science that studied how people intuitively reason about programs. In particular, Correa et al. (2023), Ho et al. (2018), and Sanborn et al. (2018) used a simple programming game to study how people trade off “Action Efficiency” against “Representation Efficiency”. Action Efficiency is a behavioral metric of how efficiently the agent achieves its goal—in this case, the number of steps or actions that it takes for a program to solve an input problem. This is a computational formalization of traditional psychological theories (Csibra, 2008; Gergely & Csibra, 2003) of agents as entities that act towards achieving some goal, moving efficiently to minimize effort. Representation Efficiency measures the complexity of an algorithm, for example, the number of lines of code or minimal description length. It formalizes an Occam’s Razor assumption of simplicity bias, where a simpler program is more representationally efficient, similar to theories in machine learning (Jefferys & Berger, 1992) and theoretical computer science (Li & Vitányi, 2008). Our experiment design and metrics are similar to those of Sanborn et al. (2018), except that in their experiments, participants actively construct programs to solve tasks, whereas in ours, participants observe the programs of other agents.

Separate from, but related to the work just discussed on the evaluation of programs, is research on the attribution of intelligence, part of a broader program on the attribution of competence (e.g., Kun & Weiner, 1973; Legg & Hutter, 2007; Mackie et al., 1990; Surber, 1984). Much of this work studies how features, cues, and prior beliefs interact to lead to an attribution of intelligence or competence, rather than a generalized mechanism that considers the underlying computations that drive behavior. Recent work also examined such attributions from first principles (Kryven et al., 2021), teasing apart planning-based and action-based evaluations. However, that work relied on assuming the general format of the computations that participants attributed to others and then inferring the specific deviations from a best plan, rather than giving participants access to the computations directly as we do here.

### Evaluating Agents in AI: The Turing Test and the Blockhead

While our main question is how humans intuitively evaluate the programs that drive the actions of agents, our work also echoes a longstanding research program examining the evaluation of machine minds, going back to the Turing Test. In the classic version of the Turing Test (Turing, 1950), human judges have a text conversation with an agent, and are asked to decide whether the agent is a human or an artificial agent. If a sufficient proportion of judges cannot correctly distinguish between the human and the machine, then the machine passes the test. With some oversimplification, the Turing Test (Turing, 1950) follows the behaviorist approach in psychology and also echoes arguments from earlier philosophical debates about mind-body dualism (Descartes, 1641) and materialism, e.g.: *“If there was a parrot which could answer every question, I should say at once that it was a thinking being”* (Diderot, 1746).

Many objections have been raised to the Turing Test (see e.g., Downey, 2014; French, 2000), including the argument that the test alone is insufficient to replace the question of whether machines think. In particular, Block (1981) argued that the Turing Test is insufficient as it does not account for the algorithms the machine uses to pass the test. To make this point, Block imagined a theoretical gigantic lookup table (the *Blockhead*) that stores the first few minutes of all likely routes in a conversation. Behaviorally, the Blockhead is identical to a complex agent with a rich world model and sophisticated understanding of language. However, if people intuitively consider such a lookup table to be less ‘intelligent’ than the complex agent, then the Turing Test must be augmented to include the algorithms that achieve a given behavior.

In contemporary AI research, the Turing Test remains an important conceptual benchmark and touchstone for the evaluation of human-level intelligence in artificial agents (Elkins & Chun, 2020; Nov et al., 2023). The arguments of Turing and Block are often echoed in modern scientific and philosophical discourse about Large Language Models (LLMs) and other neural network models (e.g., Jones & Bergen, 2023; Srivastava et al., 2022; Zhao et al., 2023). Engineers and scientists who study artificial agents often find themselves in a situation similar to our opening example, but without access to the underlying program that is driving an agent’s behavior. This is particularly the case when it comes to LLMs and their variants (Zhao et al., 2023). There is much debate in contemporary AI research as to whether such an agent has ‘truly’ learned to solve various tasks, or whether it has merely learned a large repertoire of memorized responses (e.g., Bender et al., 2021; Bubeck et al., 2023). This debate fuels the current enterprise of AI interpretability (Bereska & Gavves, 2024; Castelvecchi, 2016; Gilpin et al., 2018; Zhang et al., 2021), as well as alignment research (Ngo et al., 2022), which both presuppose that it does matter what algorithms are implemented by an agent, beyond behavioral performance. Our work here with people is motivated in part by the classic and modern arguments on evaluating machine minds, and our work also contributes to this debate by showing whether and how people intuitively care about the programs that drive behavior.

### Block Programming

In our experiments, we used a simple block programming language inspired by educational programming languages. These languages were developed to help children and other people who have never programmed before understand basic concepts. Our use of these programs follows previous cognitive science work that represents mental concepts as programs (Correa et al., 2023; Rule et al., 2020; Sanborn et al., 2018; Ullman & Tenenbaum, 2020).

Logo (Papert, 1980) and Karel (Pattis, 1994) are two well-known educational block programming languages for constructing programs that control an agent to move in two-dimensional space. For example, a Logo program might tell an agent to draw a triangle by the following steps: *move forward*, *turn left 120 degrees*, *move forward*, *turn left 120 degrees*, *move forward*. A useful innovation of these languages is the development of visual programming interfaces that enable people to program without dealing with text code. Block programming languages such as Google Blockly (Fraser et al., 2021) and MIT Scratch (Resnick et al., 2009) represent code primitives as *blocks*. Code blocks, sometimes compared to LEGO blocks, have sockets and holes that show a learner how primitives in the language can be composed by utilizing preexisting visual affordances. Block programming (and other visual programming interfaces) help children and adults to understand and write programs in mere minutes, even if they have never seen computer code before. Such tools can thus be used to examine cognition in complex, structured domains, without requiring prior programming experience that may bias participants towards coding principles they explicitly learned while programming (e.g., *“don’t repeat yourself”*).

Our experiments aimed to test whether people value the programs driving agents, and not only their behavior. This is an empirical study motivated by the Blockhead objection to the Turing Test, which aims to examine whether people intuitively evaluate agents based only on their behavior, or whether they also value the algorithms driving that behavior. We used a simple block programming language with a total of 10 unique block types, where programs control agents to navigate mazes. Within 10 minutes, we taught online participants to understand programs in this language, including a majority of participants who have never programmed before. Beyond examining whether people intuitively evaluate agents based on the algorithms that drive their behavior, we examined which features of the program or algorithm specifically matter for this evaluation. In the following section, we provide formal details of our three metrics for agent programs: Action Efficiency, Representation Efficiency, and Generalization. We then describe our experimental setup, including the details of our block programming language. Finally, we present results for our two experiments, where we find that participants value all three metrics, including evidence that people intuitively prefer programs that solve more mazes, even when given no instruction to do so.

## PROGRAM EVALUATION METRICS

We propose three metrics of program evaluation: Action Efficiency, Representation Efficiency, and Generalization. Action Efficiency and Representation Efficiency are inspired by Sanborn et al. (2018), as well as other work in cognitive science and artificial intelligence. We also propose a new metric, Generalization.

**Figure 2.**
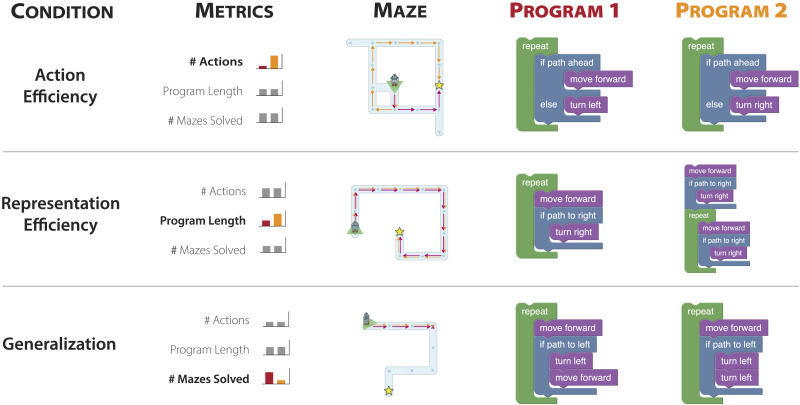
**Sample programs and mazes for Experiment 1.** This shows one example stimulus triplet (Maze, Program 1, Program 2) for each of our three experimental conditions. Each pair of programs differs by only one metric according to the condition, and the programs are equal across the two other metrics. For example, in the Action Efficiency condition, both programs have equal Representation Efficiency and Generalization, but one program reaches the goal faster than the other. In the Action Efficiency and Representation Efficiency examples, both programs succeed in reaching their goals. In the Generalization example, both programs fail to reach their goal after following the same sequence of actions—moving forward until they hit a wall. In each pair shown, Program 1 is preferred according to the metric for that pair’s condition. Left-right presentation ordering was randomized in the experiments.

In our experimental paradigm described in Program Evaluation Metrics, each test trial contains two programs that solve, or fail to solve, the same maze. Our experiment used six conditions, with three trials for each, for a total of 18 test trials. To help compensate for our metrics’ implicit assumptions about the structure of maze hypothesis space, we selected contrasting examples for our experiments that have orders of magnitude differences in our ad-hoc computed generalization metric. Test program pairs were designed so that the two programs are equivalent according to two of our metrics and only differ in the third metric (see Figure 2 for examples). These three program evaluation metrics are described below.

### Action Efficiency

A basic assumption of many theories of agency in cognitive science and reinforcement learning in AI is rational action. For example, all else being equal, an agent should prefer a shorter path to their goal over a longer path (Sutton, 1988). In discrete agent models such as Markov Decision Processes, this corresponds to minimizing the number of actions that an agent takes to achieve maximum reward (Bellman, 1957). In the maze domain, many different paths can lead to the same goal, and different programs can solve the same maze more or less efficiently. Relative to a given maze *m*, faster programs will have higher Action Efficiency. Action Efficiency is a purely Behaviorist metric, since it can be assessed based on observing an agent solve a maze, without seeing the program that generated that behavior.

$$\text{Action}\;\text{Efficiency}\left(p,m\right)=-\left|\text{Run}\left(p,m\right)\right|$$

where *Run* is a function that executes a program *p* on a maze *m*, and returns the list of actions that the program takes. Following Sanborn et al. (2018), we define Action Efficiency (A.E.) by the negative number of actions the robot takes to reach the goal. In maze navigation videos, each action the robot makes, including movement and conditionals, takes an equal amount of time, so Action Efficiency is proportional to video length.

### Representation Efficiency

Representation Efficiency of programs is determined by properties of the program’s code representation, independent of its functionality. The simplest complexity metric comparing two programs is the total number of lines of code, or the number of nodes in the program expression graph. This is related to the concepts of minimal description length and Kolmogorov complexity in algorithmic information theory (Li & Vitányi, 2008). Such simplicity priors are common in AI (Jefferys & Berger, 1992) and statistics (Schwarz, 1978), and are even argued to be a fundamental property of cognition (Chater & Vitányi, 2003; Goodman et al., 2008; Ullman et al., 2012).

$$\text{Representation}\;\text{Efficiency}\left(p\right)=-\left|p\right|$$

In our case, we define Representation Efficiency as the negative number of blocks used in a single program *p*, the negative code length −|*p*|. This can be thought of as an extension of Occam’s razor, the preference for simpler hypotheses—programs—over more complex ones. Representation Efficiency is analogous to a traditional, albeit flawed, measure of text program complexity, which is commonly used in practice: the number of lines of code in a file (Jay et al., 2009). While many other possible metrics could also measure program complexity, for example, how deeply functions are nested inside each other, or the number of unique functions used, Ho et al. (2018) found that human behaviors are adequately explained by the simple measure of program length, compared with a variety of alternative representational metrics.

### Generalization

Action Efficiency is a purely behaviorist metric that measures how well an agent performs a task. Representation Efficiency evaluates the program, while ignoring its behavior. But beyond these considerations, people may evaluate how well an agent would do on alternate tasks, beyond the one provided. An intelligent agent doesn’t merely do one task or memorize answers to specific questions, it solves a wide array of tasks. To examine this, we present a novel program evaluation metric, Generalization: how well a program or agent performs, on average, over a space of possible inputs. We stress that ‘generalization’ has, of course, been studied in many different domains within and across AI and cognitive science. We mean that our *metric* is novel in studying whether people intuitively evaluate the generalization of an agent’s program.

Generalization is both a behavioral metric, like Action Efficiency, and a cognitive metric, like Representation Efficiency. The Turing Test similarly aims to test an agent’s behaviors in a domain where a wide variety of sub-tasks can be tested − conversation. However, following the Blockhead objection, if a human judge observes the algorithm driving an agent, it might change how they perceive the agent’s Generalization abilities. Our experiments test this hypothesis, using a single example of behavior for each agent, so that observers must estimate generalization based on the agent’s algorithm.

In our experiments, Generalization is defined as the fraction of mazes solved by the program, where a program that solves more mazes is said to ‘generalize better’ than one that solves fewer mazes:

$$\text{Generalization}\left(p\right)=\frac{1}{\left|M\right|}\sum_{m\in M}\text{Solves}\left(p,m\right)$$

**Figure 3.**
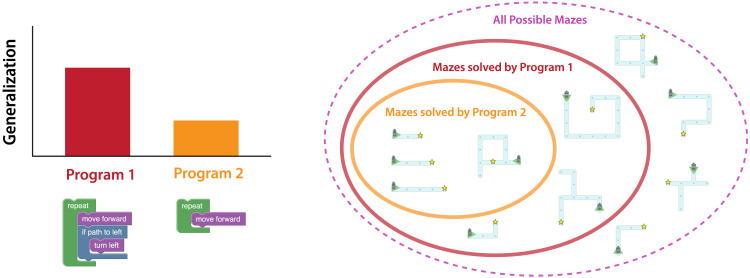
**Generalization measures the number of different tasks that an agent can solve.** We estimate Generalization as the fraction of all possible mazes where a given program leads the agent from its starting position to the goal. (Left) In this example, 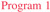 has higher Generalization than 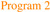. (Right) This is because Program 2 solves more mazes than Program 1. In addition to the straight paths to the goal that Program 1 solves, Program 2 also solves many other mazes that involve left-turning paths. Neither program solves mazes with right-turning paths to the goal.

Each maze navigation program solves a different set of mazes, and for some programs, the set is much larger than others. Generalization measures how well a predictor generalizes across samples in a distribution, as the proportion of all mazes that the program solves (Figure 3).

For example, in a test pair for the generalization condition (Figure 3), Program 1 will solve any maze that involves only forward movement and right turning, including complex mazes with spiral paths, junctions, and alternate dead ends. The code for Program 2 is nearly identical, but this program never follows rightward paths since it only ever turns right twice in a row, and only solves mazes with a straight-line path to the goal. Program 1 will solve any maze that Program 2 can solve, and many more.^1^ Thus, Program 1 will have higher Generalization than Program 2. Generalization is a cognitivist metric in our experiments, since participants in the Generalization condition observe equal Action Efficiency behaviors for both agents, and only the program code is different.

For our experiments, described in the following sections, each participant saw each program only once in the experiment, paired with a single data point of behavior on one maze. This let us avoid showing a participant the same program’s behavior on multiple different mazes, which might bias participants to use Generalization as a feature for what makes a “better” program. Additionally, we estimated Generalization by randomly generating a set of mazes *M* and computing the fraction of those mazes solved by a block program. The number of unique mazes is exponential in maze size (2^W*H^ for a maze of size *W* × *H*), so we approximated the space of mazes by sampling *M* mazes. We used Prim’s algorithm (Prim, 1957), a famous graph algorithm in computer science commonly used for maze generation. Mazes generated by Prim’s algorithm do not have loops, so we modify generated mazes by randomly adding paths to a small fraction of non-path locations. Though not shown here, other maze generation algorithms were tested with similar results.

## EXPERIMENTS

**Figure 4.**
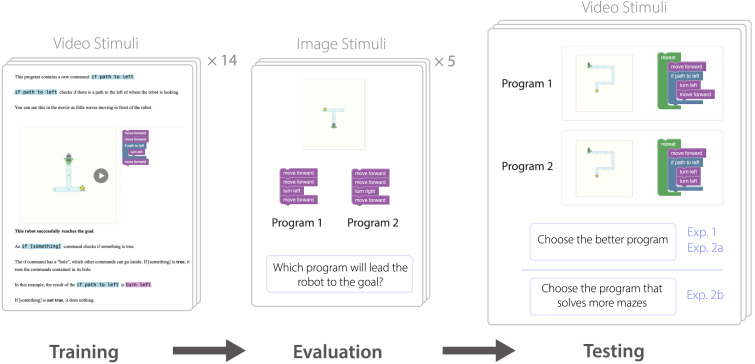
**Our experimental setup has three phases: training, evaluation, and testing.** In training, participants learn to program in our simple programming language, with steps to introduce each programming primitive. In evaluation, we assess whether participants have successfully learned the task by testing their performance on 5 simple tasks with one correct answer. In testing, we observed how participants respond to questions with ambiguous answers: *‘Choose the better program’* or *‘Choose the program that solves more mazes’*.

We conducted two experiments, both with very similar designs. Both experiments had three sections: training, evaluation, and testing (Figure 4). The stimuli in the training and test phases of the experiments were videos of a maze and block program side-by-side, similar to Figure 3. These videos were approximately 5–30 seconds in length. As the program runs, each block is highlighted when it triggers, and the robot follows the corresponding command. For example, a “move forward” command would highlight and the robot moves a step forward, then the next command would highlight, and so on. When “if” commands execute, a short animation follows where a few waves radiate out from the robot in the specified direction.

During training, participants saw a series of 14 pages, each with example videos as well as accompanying text. The text introduced new commands that the participant was seeing for the first time, describing the behavior of the command, and emphasizing important information in the video to notice. Some of the training examples included program failures as well, so participants saw what happens when a program does not lead the robot to its goal. These failure trials showed examples where an agent tried to move into a wall, which caused the program to immediately stop running.

In the evaluation phase, participants saw 5 examples with static images of mazes and programs. A single maze was shown with two different programs, where only one of them actually solves the maze. These examples are fairly easy but require an understanding of our block programming domain and the operation of maze navigation programs.

For testing, participants saw a sequence of 18 trials in Experiment 1 and 15 trials in Experiment 2. In each trial, participants were shown two videos of two different programs that either both solved or both failed to solve the same maze. In Experiment 1, participants were instructed to *Choose the better program*, with no further instruction of what “better” should mean. Experiment 2 is identical to Experiment 1, except that it includes additional test videos, as well as an additional task condition where participants were instead instructed to *Choose the program that solves more mazes*.

All data and stimuli for Experiments 1 and 2 are available on OSF^2^.

### Block Programming Language

Our experiments used a simple block programming language implemented in Blockly (Fraser et al., 2021), which is similar to Scratch (Resnick et al., 2009), an educational programming language used to teach programming to kids. With this framework, we were able to teach people with no programming experience how to program in our simple domain in a short span of time (15–20 minutes).

The programming language we used has 7 different block types. There are 3 movement blocks: move forward, turn left, and turn right, and 4 blocks that take other blocks as arguments: if path to right, if path to left, if path forward, and repeat. Block commands are arranged vertically in sequence and, as in any imperative programming language, executed sequentially one after the other.

The conditional if blocks and repeat block accept other blocks horizontally as arguments in a block-shaped visual hole. Conditional blocks execute code in the first block if the condition is true, for example if there is a path directly in front of or to the right of the robot, and also optionally include a second “else” hole that runs commands only if the condition is false. The Repeat block repeatedly executes the blocks it contains until the agent reaches the goal.

### Experiment 1

Experiment 1 evaluated whether participants would show preferences according to our three metrics. It was also a demonstration that online participants, notably those with no programming experience, were able to learn our task sufficiently well enough to have the ability to use algorithm-only features in their evaluations of what makes a program “better”.

**Figure 5.**
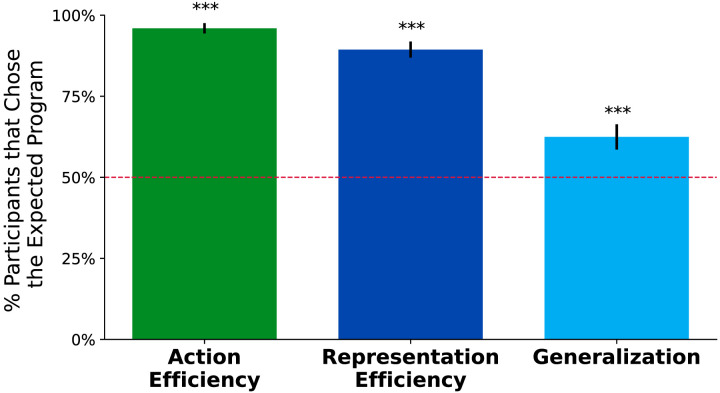
**Experiment 1 responses by condition.** Proportion of participants in Experiment 1 who chose the program we predicted, across three conditions corresponding to our three metrics: 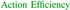, 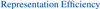, and 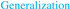. Error bars correspond to 95% confidence intervals for a *z*-test of a binomial proportion. Across all conditions, participants preferred programs that are more efficient or generalize better according to our metrics. As predicted by a behaviorist theory of rational action, in the Action Efficiency condition people prefer agent programs that solve the maze faster. Our results in the Representation Efficiency and Generalization conditions support our theory that people are intuitive cognitivists when assessing agents.

Experiment 1 had three trials for each of three conditions: Action Efficiency, Representation Efficiency, and Generalization. These three conditions were designed such that only the metric for that condition would vary while the other two were kept fixed, e.g., each trial in the Action Efficiency condition had much slower videos in one program than the other, but equal Representation Efficiency (program length) and Generalization (Figure 5).

We also collected data for three trials in each of three ‘crossed’ conditions, which we do not analyze here. These conditions traded off two metrics against each other, while keeping the third metric fixed. We did not pursue this line further, as in Sanborn et al. (2018), due to the challenge of comparing units. For example, how many blocks of code (Representation Efficiency) will people trade against a single action (Action Efficiency)? Answering this question would require further experiments to determine psychometric curves, and for this reason, we do not analyze these trials.

#### Participants.

In the first experiment, we collected data from 198 participants from Prolific. The number of participants *N* = 200 was chosen based on a power analysis, which showed that a modest effect size of *μ* = .6 will have a *p*-value < .001 with a two-sided Binomial test against *p* = .5 (i.e., no effect). 200 participants were collected, 1 data point was lost due to collection error and 1 participant was excluded due to fast (<3 sec.) response times in test trials.

60% of the remaining participants reported no prior experience with programming, but still correctly answered 95% of the evaluation questions. Participants with programming experience correctly answered 96% of evaluation questions. We coded participant responses with clear binary responses (e.g., “No, I’ve never coded before”, “I took a college course on Python”) as 0 or 1 and coded marginal responses (“Basically no programming experience”, “only some HTML”) as 0.

#### Results.

Analyzing each test trial as an individual experiment with *N* = 199, we used Binomial testing with Bonferroni correction (*α*/*m* = 0.005/6) to find results are significantly different from *H*_0_ = 0.5 (Figure 5). All trials were significant in all three conditions. Participants vastly prefer programs that reach the goal faster, or are more Action-Efficient, all else equal (*μ* = .96, *p* < .001). Programs that have shorter code representations, or are more Representation-Efficient, are also vastly preferred (*μ* = .89, *p* < .001). Participants also prefer programs that solve more mazes, or Generalize better (*μ* = .62, *p* < .001).

### Experiment 2

In Experiment 1, we established our methodology of Block programming as a tool for assessing people’s evaluation of agents, and found that people were sensitive to all 3 metrics we considered. In Experiment 2, we had two goals: first, to verify that our findings are robust, by replicating the results of Experiment 1 with a much larger stimulus set. Second, to test whether people specifically use our measure of generalization—the number of mazes that a program can solve—when assessing program goodness. Experiment 2 used an identical experimental setup to Experiment 1, except with 60 unique testing stimulus pairs (20 per condition) compared with only 9 analogous stimulus pairs (3 per condition) in Experiment 1. Each participant in Experiment 2 saw a random subset of 15 stimulus pairs out of 60, after pilot testing showed effects of mental exhaustion after 15–20 trials. Participants were split into two groups, where in the *Better* condition they saw identical prompts and questions as in Experiment 1 (“Please choose the better program”), and in the *Solves* condition they were instead prompted: “A single program may be able to many different mazes, beyond the single maze shown in the video … Please choose the program that solves more mazes”. The experimental design and analyses for Experiment 2 were pre-registered on AsPredicted^3^.

#### Automatic Stimulus Generation.

It is difficult to manually generate test stimulus programs and mazes that are equal in two of our metrics but significantly different in the remaining one. This led us to use automatic stimulus generation for Experiment 2. We used a probabilistic context-free grammar implementation of our Block Programming Language to randomly sample hundreds of thousands of unique navigation programs, and filtered programs to only those with no more than 8 blocks, not counting a default Repeat block. As with estimating Generalization, we used Prim’s algorithm with random loops to sample random mazes. Test stimuli were determined by iterating through pairs of sampled programs and mazes, to automatically find stimuli that were equal (or nearly equal for Generalization) in two of our metrics and substantially different in the third metric. Both programs either succeeded or failed at solving the maze. Our generation method was unable to create failure pairs in the Generalization condition.

#### Participants.

We collected data from 400 participants on Prolific. 2 participants were excluded due to very fast response times (<5 min.), and 2 additional participants were collected to compensate. Stimuli were randomly selected and balanced across participants, totaling approximately 50 responses per stimulus pair and question condition. The median experiment time was 25 minutes, and participants were compensated $4.50 for their work. As with Exp. 1, the number of participants *N* = 400 (*N* = 200 each for the *Better* and *Solves* conditions) was chosen based on a power analysis which showed that a modest effect size of *μ* = .6 will have a *p*-value < .001 with a two-sided Binomial test against *p* = .5 (i.e., no effect) with *N* = 200.

Before running the analysis, we filtered data from low-effort participants and those who failed attention checks. Following our preregistration, we excluded data from participants who failed an explicit attention check question at the end of the survey, as well as those who got 2 or more of the 5 evaluation questions wrong. After collecting data, we noted that a number of participants did not spend enough time on parts of our experiment to have fully watched the stimulus videos. Although this was not pre-registered, we further excluded data for participants whose total experiment time was less than 10 minutes or whose median response time per test trial was less than 10 seconds (the median of video time for each stimulus was 23 seconds). After exclusions, this left 323 participants for our analysis. Finally, another point that we considered after data collection is that failures in the Action Efficiency condition are likely interpreted in a different way from successes. Following prior work, our Action Efficiency metric is defined as the number of steps an agent takes to reach its goal, but our model does not explain how people evaluate agents that more efficiently *fail* to reach their goals. We keep failures in the Representation Efficiency condition since that metric does not depend on behavior.

We coded programming experience in the same way as in Experiment 1. 60% of participants in Experiment 2 reported no prior experience in programming, but still answered 94% of evaluation questions correctly. Participants with programming experience answered 99% of evaluation questions correctly.

#### Results.

**Figure 6.**
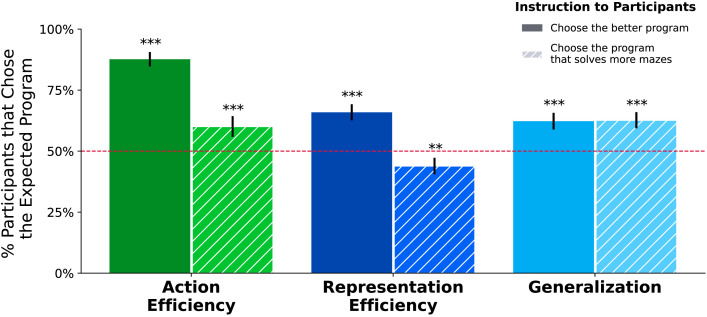
**Experiment 2 responses by condition.** Experiment 2 replicates Experiment 1 with additional stimuli—20 per condition instead of 3—and adds an additional condition for whether participants were instructed *Choose the better program* (‘Better’, solid color bars) or *Choose the program that solves more mazes* (‘Solves’, striped bars). Error bars correspond to 95% confidence intervals for a *z*-test of a binomial proportion. With the *Better* instruction, we find similar results as Experiment 1. Responses for the *Solves* instruction are nearly identical to the *Better* instruction in the Generalization condition, whereas Action Efficiency and Representation Efficiency conditions are non-significant or go in the opposite direction for *Solves*. This result supports our hypothesis that people are indeed judging which program solves more mazes, even when they are *not* instructed to do so.

As Figure 6 shows, in the *Better* condition, participants greatly preferred the more Action Efficient, Representationally Efficient, and the higher Generalization programs. The fraction of participants *μ* who chose the same program as we predict for each condition, as well as the *p* value for a two-sided Binomial test against *P* = .5, with Bonferroni correction treating each of the 6 (3 × 2) conditions as an independent experiment, are listed in Table 1. In the *Solves* task, participants’ preference for faster programs and shorter code decreased substantially for the Action Efficiency and Representation Efficiency conditions compared to the *Better* task, which is to be expected since participants were instructed to pick the program that solves more mazes, which may not be the program that’s *better*. In the Generalization condition, however, we see that the preference for the higher Generalization programs is nearly identical across both question groups. This is reflected in the Fisher *p* values—an analysis which we added after pre-registration—which show significant group differences between the *Solves* and *Better* tasks for the Action Efficiency and Representation Efficiency conditions, but not for Generalization.

**Table 1.** **Experiment 2 mean values and Binomial significance tests.** Experiment 2 had a 3 × 2 × 2 design across three conditions (Action Efficiency, Representation Efficiency, Generalization) and two instructions *Choose the better program* (‘Better’) vs. *Choose the program that solves more mazes* (‘Solves’). *μ* is the fraction of participants who chose the same program as we predict, and Bonferroni-corrected (*m* = 6) *p* values are for two-sided Binomial tests against a random baseline *P* = .5. Also see Figure 6.

**Condition**	**Task**	*μ*	Binom. *p*	Fisher *p*
Action Efficiency	Better Program	.88	<.001	<.001
	Solves More Mazes	.60	<.001	
Representation Efficiency	Better Program	.66	<.001	<.001
	Solves More Mazes	.44	<.005	
Generalization	Better Program	.62	<.001	.97
	Solves More Mazes	.63	<.001	

**Figure 7.**
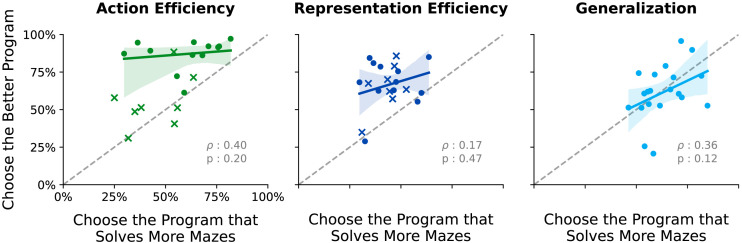
**Experiment 2 correlation between *Better* and *Solves* conditions.** Correlations for each test trial in Experiment 2, between the fraction of participants who selected a program with the prompt *Choose the better program* and the fraction who chose the same program with the prompt *Choose the program that solves more mazes*. Circles: trials where both programs succeeded. Xs: trials where both programs failed. Note that for the Action Efficiency condition, correlations omit failures (Xs), whereas correlations include failures in the Representation Efficiency condition.

Finally, we examined Spearman correlations between trial-by-trial average responses across the *Better* and *Solves* groups (Figure 7, Table 1). In this analysis, each of 60 data points corresponds to a stimulus pair of two programs running on the same maze. Each datum has two values: the fraction of participants who chose Program 2 in the *Better* condition, and the fraction of participants who chose Program 2 for the same stimulus in the *Solves* condition.

## DISCUSSION

In Experiments 1 and 2, we find that participants prompted to choose the “better” program reliably prefer programs with faster run times (Action Efficiency condition), programs with shorter code (Representation Efficiency) and programs that can solve more mazes (Generalization). For all conditions, when instructed to choose the better program, the fraction of participants that chose our expected program is significantly greater than chance (*μ* > .5) by a Binomial test. Experiment 2 replicated the findings of Experiment 1, with 20 unique stimuli per condition rather than 3, as well as testing the original prompt *Choose the better program* with *Choose the program that solves more mazes*. In Experiment 2, we find similar results to Experiment 1 (*μ* > .5) for all conditions in the *solves more mazes* task. More specifically, when comparing the *solves more mazes* task to the *choose the better program* task, we found that the two tasks produce similar results for the Generalization condition, but show different results for the Action Efficiency and Representation Efficiency conditions. This is in line with our hypothesis that when people are asked to choose the better program for stimulus pairs in the Generalization condition, they evaluate the Generalization of the programs by estimating which program solves more mazes. Further, when people were explicitly told to choose the program that solves more mazes, they chose the higher-generalization program at an equal rate to when they were instructed to choose the better program. This suggests that participants in the *Better* condition may be close to their maximum performance in this experimental setting, given the available training and performance incentives.

A number of other findings in Experiment 2 are also noteworthy. That we find non-significant correlations between the *Better* and *Solves* instructions suggests that people are not using the same metrics to evaluate programs (or agents) across these two tasks. For the Action Efficiency and Representation Efficiency conditions, this is not surprising, since we do not expect Generalization to play a major role in these trials when people are instructed to choose the better program. We expect that people first estimate the number of actions and code length before resorting to estimating program generalization, since the former metrics likely require much less cognitive effort. For the Generalization condition, however, this result was surprising. We predicted to find a stronger correlation in the Generalization condition between which programs people thought were better, and which ones people estimated to solve more mazes. This lack might be because people’s intuitive method for estimating Generalization is not to specifically estimate which program solves *more* mazes. For example, people may intuitively estimate a related but different value, such as ‘which program solves *more complicated* mazes’. Beyond this, we found that people estimate that programs which solve a maze faster (Action Efficiency condition) and which have longer code (Representation Efficiency condition) will also solve more mazes. This may suggest that when people fail to notice a difference in the Generalization of two programs - the number of mazes each can solve - people may use the program’s Action or Representation Efficiency as a fallback approximation. However, for now, these suggestions are speculative, and further studies on how people estimate the generalization of programs would be useful.

Inspired by the philosophical Blockhead objection to the Turing Test, we aimed to test empirically whether people are Intuitive Behaviorists who evaluate agents based only on their behavior, or Intuitive Cognitivists who evaluate agents based on the algorithms driving their behavior. Our results support the idea that people are Intuitive Cognitivists, since people prefer programs that have shorter code and which can solve more mazes, even when both agents behave in the same way. In Experiment 2, we find that people give similar responses for *Choose the better program* and *Choose the program that solves more mazes* for the Generalization condition only, and different patterns of responses for the Action Efficiency and Representation Efficiency conditions. This suggests that people are indeed estimating something like *how many mazes could this agent solve?* when deciding which program is better.

However, we do not expect that people are actually considering hundreds or thousands of different possible mazes, as we do in our algorithm. Instead, we expect that people estimate generalization by efficiently searching over a small number of representative data examples which approximately characterize a program. This problem presents an exciting direction for future work, to understand the cognitive processes that enable people to estimate program generalization. A related question is how people represent and reason about programs when they are shown explicit code, and what happens when people hit their limits of comprehension. This is particularly relevant to the Generalization condition and “Solves More” instruction in our experiments, since estimating the number of mazes a program solves requires reasoning about what the program does. One way that people might reason about programs would be to systematically simulate each step in a program’s execution, and consider whether the program solves a maze. However, with sufficiently complex programs or mazes, this sort of mental simulation is likely to become infeasible. It seems likely that, for many of the programs in our experiments, people did not rigorously mentally simulate the full execution of the programs. Instead, we speculate that people might intuitively represent the behavior of chunks of long programs—e.g., *“this program moves forward, it can turn left, and sometimes it turns right”*. People might represent mazes and other input data with similar high-level descriptions, such as *“left-turning spiral”* or *“zig-zag from the bottom-left to top-right”*.

Our results in the Action Efficiency condition demonstrate that people intuitively evaluate agents based on their behavior, in line with prior work in cognitive science showing that humans expect agents to take efficient paths to reach their goals (Baker et al., 2009, 2017; Gergely & Csibra, 2003; Jara-Ettinger et al., 2020). In the Representation Efficiency condition, we find that people prefer shorter programs, similar to prior work suggesting that people have a bias to prefer simpler concepts with shorter description length (Chater & Vitányi, 2003). We also find a bias where people estimate that longer programs can solve more mazes, similar to related work which studies cases where people have a complexity bias, rather than a simplicity bias (Dubova et al., 2025). The Generalization condition follows a guiding principle of AI and machine learning, to develop algorithms that solve a range of problem instances (out-of-distribution generalization) and are generally intelligent enough to solve a variety of different problems (Legg & Hutter, 2007; Russell & Norvig, 2010).

Testing people’s intelligence attributions based on cognitive/behavioral mechanisms poses several key challenges for generalizing the conclusions of our studies. First, in our experiments, we presented code directly to participants as *cognitive* features, compared with the *behavioral* features of agent actions. But in everyday real-world scenarios, people don’t directly observe the program driving an agent’s behavior. The Turing Test emphasizes this aspect of behaviorism: if an agent is behaviorally indistinguishable from a human at some task, it must be capable of doing that task. The test operationally *replaces* the question of whether a machine can ‘think’. Even if people are intuitive cognitivists, presumably they must infer a program driving behavior first, and then evaluate that program. Our tests do not account for the first part of this process. Then again, in the real world, people also don’t usually observe an agent’s *behavior* on vast arrays of situations in order to make their judgments about another agent. Instead, we often use prior knowledge acquired through learning and genetic endowment to make sense of extremely limited data (Ullman & Tenenbaum, 2020).

Another limitation of our design is that we directed participants’ attention towards the algorithm driving agents. It is possible that our experimental framing biases participants towards choosing agents based on features of their underlying algorithms, since much of the content in our experiments is about programs. Then again, it is unclear how to effectively test the Blockhead objection without introducing such a framework, since any experiment broadly following its contours would need to display machine behavior alongside algorithms or programs that dictate that behavior. Then again, it is still possible to object that by specifically asking participants to evaluate *programs* that are driving an agent’s behavior rather than the *agent* itself, we are missing some underlying dissociation between agents and programs that would meaningfully alter participant answers^4^. To this, we note that in the original Blockhead thought experiment, there is generally an equivalence between machines and programs that are driving their behavior, and in our experiments as well, there is a one-to-one correspondence between behavior and programs. However, to address this point more empirically, we pre-registered and conducted a study similar to Experiment 2, but replacing the evaluation of ‘programs’ with the evaluation of ‘agents’. The details of this Experiment (S1, *N* = 300) are in the Supplementary Materials, but briefly, we found that across all conditions, people prefer the expected program greater than chance, and we found no significant differences between people’s preferences in the conditions which evaluated cognitive metrics (Representation Efficiency and Generalization Efficiency). These results support our claim that our experiments indeed test people’s evaluation of agents more generally, and go against the view that our phrasing, such as *“Choose the better program”*, may have inadvertently biased participants towards evaluating the ‘programs’ instead of the ‘agents’.

While we found that people evaluated the goodness of programs driving agent behavior on the basis of several different features, it may be objected that this is not the same as attributing intelligence to the agent, but rather that it is an attribution of intelligence to the programmer. To put it plainly, when seeing a particularly clever program P, one might think that the person who designed P is clever, rather than thinking that the machine that runs P is clever^5^. We note that this issue parallels an objection noted as Objection 1 in Block (1981), in which the intelligence (or lack thereof) of a program is attributed to the programmer. Paralleling Block’s reaction to this, consider that if one were to converse with a human-like machine which made a clever remark, and if its conversational ability was based on code that was also human-like (including for example general learning principles, clever hacks, generalizable planning algorithms, context-relevant switching, and so on), then the cleverness of the remark could be attributed to the machine itself. If instead we were to find the remark was simply the result of a pre-programmed look-up table, then we would rightly attribute the cleverness to the programmer. It is the structure of the program leading to the behavior that entitles us to make these attributions to the machine or the programmer. On top of this, one could in *principle* credit the programmer for the overall design of the program, but it would be similar to saying ‘the programmer isn’t actually clever, it is their biological evolution and their education that gave them these abilities’. Rather, one might marvel at evolution and education, while still crediting the programmer for their work—that is, unless the programmer’s own behavior is in turn driven by programs that are like look-up tables.

The majority of participants in our experiments reported having no prior programming experience, and although we found similar results across programmers and non-programmers (Supplementary Materials), participants with programming experience might have explicit, learned biases about what constitutes a “good” program, such as using fewer lines of code. A more naturalistic experiment design might use natural language instead of computer programs to represent algorithms. However, this would require additional experimental design to map language to algorithms and to ensure that language was interpreted by participants as algorithms driving agents’ behavior rather than post-hoc explanations.

Our work highlights an important interaction between concept simplicity, or Representation Efficiency, and Generalization. Many hierarchical cognitive models assume that people possess an innate simplicity bias (Chater & Vitányi, 2003; Ho et al., 2018; Sanborn et al., 2018), and prefer stimuli with shorter description lengths. However, as we encountered in designing stimuli for our experiments, even in very simple domains, it is difficult to come up with program pairs that are alike in all metrics except for program length. In the maze domain, when program length is the only difference between two programs with equal actions and generalization, the longer program usually has unnecessary, obviously redundant code (see Figure 2). Simplicity bias may be interpreted as a feature of resource constraints, where longer, more complex programs require more time or effort to mentally simulate. An alternative is that simplicity may be a *cue* to generalization. For example, the highest generalization programs in our experiments are simple ‘wall follower’ algorithms that can be expressed in as few as 5 code blocks^6^. These simple wall follower programs will solve any maze without loops by systematically exploring every path in the entire maze. More complicated programs are no more powerful, and additional code blocks might even decrease generalization or make the same algorithm harder to understand. Further, while these algorithms may have high generalization, they are also very slow to solve most mazes, and in practice, people likely make tradeoffs between metrics such as Generalization and Action Efficiency. An exciting direction for future work may be to measure how people trade off these different metrics when evaluating agents, and what specific strategies people use for evaluating programs. People might, for example, first evaluate either the Action Efficiency of an agent or its Generalization, before considering the other metric. Alternatively, people might simultaneously evaluate different metrics, giving more weight to one metric than another.

Beyond being important for cognitive theories of intelligence attribution, our work has broader relevance to the evaluation of artificial intelligence systems. The Turing Test is a common theme in contemporary AI research (Jones & Bergen, 2023; Zhao et al., 2023), where black-box performance on benchmark datasets (e.g., Hendrycks et al., 2020; Srivastava et al., 2022) remains the gold standard for evaluating most AI algorithms. Tools and methods from AI interpretability enable researchers to understand the representations learned by deep learning AI agents. Our results support this endeavor: if humans are intuitive cognitivists, rather than intuitive behaviorists, then the most effective way for humans to evaluate artificial agents might be by understanding their internal workings. When people evaluate other human agents, we bring a wealth of prior information to bear that enables us to make inferences about another person’s abilities, such as their *intelligence*. With artificial agents, we do not have such prior information, so AI researchers should take advantage of their ability to, analogous to our experiments, observe and model latent algorithms in AI systems.

The original Blockhead was a thought experiment (*N* = 1): an astronomically large look-up table that could carry out a seemingly intelligent conversation, and pass the Turing Test without counting as ‘thinking’. At the time it was proposed (1981), one might have countered that a lookup table with billions of memorized responses was wildly unrealistic. Any agent that humans come across that *seems* to be intelligent *is* intelligent, precisely because it would be infeasible to house a lookup table of that size in a reasonable-sized body. Yet, today we find ourselves in a situation where Blockheads may have been realized, in less-than-astronomical proportions, with artificial neural networks that have billions of parameters and can store more facts than parameters (Elhage et al., 2022). It is now a critical question for experts whether an AI agent has actually learned a sophisticated internal algorithm, such as a world model (Li et al., 2022), or whether it has merely learned something akin to a massive lookup table (Bender et al., 2021). But beyond academic arguments, everyday people care about the underlying computation that drives behavior. Or at least, they act like they do.

## ACKNOWLEDGMENTS

We thank John McCoy, Tamar Kushnir, and members of the CoCoDev and CCN labs at Harvard for their insightful comments.

## FUNDING INFORMATION

Experiment 2 was funded by a Hodgson Fund grant from the Department of Psychology at Harvard. TU was supported by the Jacobs Foundation.

## AUTHOR CONTRIBUTIONS

E.B.: Conceptualization; Formal analysis; Funding acquisition; Methodology; Visualization; Writing – Original draft; Writing – Review & editing. T.U.: Conceptualization; Funding acquisition; Methodology; Writing – Original draft; Writing – Review & editing.

## DATA AVAILABILITY STATEMENT

All of the materials for the main studies (including data and stimuli) are available in the Open Science Framework at https://osf.io/yzbrq/?view_only=ff89a0bb45b64e7d89ac21b105a4c261.

## Notes

^1^ Not shown in Figure 3, there is also a small number of mazes that Program 2 solves that Program 1 cannot, e.g., a straight line leading to the goal but with a left turn that leads to a dead end.^2^ OSF data: https://osf.io/yzbrq/?view_only=aa065360090944d0b3f7cf4cc625e01f.^3^ Pre-registration: https://aspredicted.org/87vr-bynt.pdf.^4^ We thank an anonymous reviewer for this point.^5^ We thank an anonymous reviewer for this point as well.^6^ For example, the following program solves more than 90% of mazes sampled by our maze generation algorithm: Repeat [IfPathLeft{TurnLeft, MoveForward} Else{TurnRight}].

## Supplementary Material


