# Supplementary material for: People Evaluate Agents Based on the Algorithms That Drive Their Behavior
Source: Open Mind (Camb). 2025 Aug 29;9:1411–30. doi: 10.1162/opmi.a.26 (PMC12435988; doi:10.1162/opmi.a.26)
Supplement: Supplementary file 1 [file opmi-09-1411-s001.pdf]

People evaluate agents based on the algorithms that drive their behavior

Supplementary Materials

Eric Bigelow & Tomer Ullman

Harvard University,
Department of Psychology

1 Experiment S1: Alternate Instruction Experiment

Our principal goal with our experiments was to test whether, when evaluating agents, people value not only the agents' behavior but also the programs driving their behavior. Experiments 1 and 2 in our main text aimed to empirically test by having participants evaluate pairs of programs, which differed either in their behaviors or in the program code. In these experiments, we instructed participants to *"Choose the better program"* or *"Choose the program that solves more mazes"*. We now consider that this phrasing may have inadvertently directed participants' attention towards the program, rather than the agent. If this were the case, this would mean that our results in Experiments 1 and 2 might not directly test the principle that people evaluate agents based on the programs driving them.

In order to test whether our main results might have been affected by directing people's attention to the *program*, instead of the *agent*, we ran an additional experiment [1]. Experiment S1 is identical to Experiment 2, except that it only has one instruction condition, where participants are instead instructed to *"Choose the better robot"* in the Test Phase of the experiment. Additionally, the stimuli in the Evaluation Phase of the experiment were modified to *"Which robot will reach the goal?"*.

Participants

We collected data from 300 participants on Prolific, who were compensated $6 for their work. 1 participant was excluded due to a very fast completion time ($<$ 5 min.), and 1 additional participant was collected to compensate. The number of participants $N = 300$ was chosen based on a power analysis which showed that with $N = 300$ is more than sufficient to ensure that an effect size of .15 will have a power of at least .8 with a Fisher test against the results of Exp. 2 (*Better* instruction only). After filtering out low-effort participants with the same method as in Exp. 2, of the remaining $N = 239$ participants, mean accuracy on the evaluation questions was 94%, and 68% of participants had no prior programming experience.

Results

We ran three analyses. First, we found that - as with Exp. 2 *Better Program* - for all three conditions, a Binomial test against chance ($P = .5$) was significant with Bonferroni-corrected $p < .001$ (Figures 1, 2). Next, we ran a Fisher-Irwin test to compare whether results for each condition are significantly different from results in Exp. 2 *Better Program.* We found a significant difference in the Action Efficiency ($p = .011$) condition, but not for the Representation Efficiency ($p = .10$) or Generalization ($p = .82$) conditions. Third, we ran a Spearman correlation analysis (Figure 3) to test for trial-by-trial correlation between Exp. S1 *Better Robot* and Exp. 2 *Better Program.* We found a significant correlation in the Generalization condition ($\rho = .84,\ p < .01$) but not in either the Action Efficiency ($\rho = -.5,\ p = .1$) or Representation Efficiency ($\rho = .06,\ p = .8$) conditions.

Discussion

Our predictions for this experiment were that results would be similar to the results of Experiment 2 with the *Better Program* instruction. Our results for the binomial tests match this prediction, and the trend is that for all conditions, people chose the expected program at a rate that was greater than chance. For the Fisher test, our results in the Representation Efficiency and Generalization condition match expectations, since these tests did not show significant differences. However, for the Action

[1] Pre-registration: `https://aspredicted.org/fn8b-mr7b.pdf`

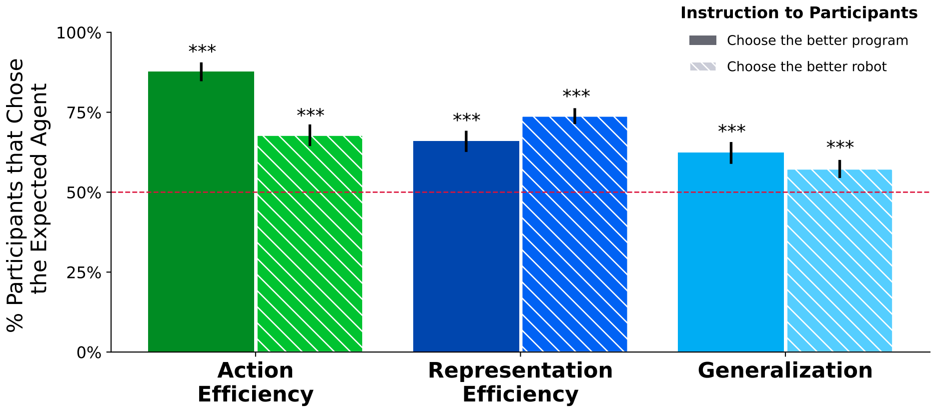

Figure 1: **Experiment S1 (*Better Robot*) responses by condition, compared with Exp. 2 (*Better Program*).** Each bar shows the fraction of participants who chose the expected program, according to our model, for each of three conditions (Action Efficiency, Representation Efficiency, Generalization), across two different instructions (*"Choose the better program"* or *"Choose the better robot"*).

Efficiency condition, we observe a significant difference. This result is somewhat perplexing: while we might have expected that the *Better Robot* instruction would either have no effect, or would lead to participants up-weighting behaviorist metrics over cognitivist metrics, instead the opposite is true. People preferred the faster agents *less* (Action Efficiency condition) when they were instructed to choose the *Better Robot*, and people also preferred the shorter programs *more* (Representation Efficiency). We may attribute the latter to sampling error, since the Fisher test did not report significant differences between groups for the Representation Efficiency condition; however, the difference between results for the Action Efficiency condition is more difficult to explain. We speculate that the *Better Robot* instruction may have led participants to attend less to the stimuli than the *Better Program* instruction, due to people having prior associations with the words *"Program"* and *"Robot"*

**Condition**	**Task**	μ	Binom. p	Fisher p
Action Efficiency	Better Program	.88	$< .001$	$< .05$
	Better Robot	.68	$< .001$	
Representation Efficiency	Better Program	.66	$< .001$	.10
	Better Robot	.74	$< .001$	
Generalization	Better Program	.62	$< .001$	.82
	Better Robot	.57	$< .001$	

Figure 2: **Experiment S1 mean values and Binomial significance tests.** Experiment S1 had an identical design to Experiment 2, but used only one instruction: *Choose the better robot* ('Better Robot'). Here, we compare results to Experiment 2 for the instruction *Choose the better program* ('Better Program'). μ is the fraction of participants who chose the same program as we predict, and Bonferroni-corrected ($m = 3$) p values are for two-sided Binomial tests against a random baseline $P = .5$. Fisher test p values (Bonferroni-corrected with $m = 3$) compare whether the results for 'Better Robot' and 'Better Program' are significantly different.

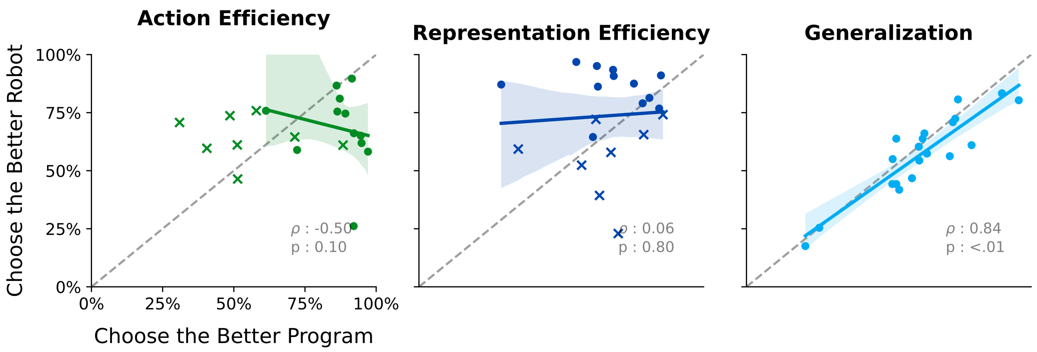

Figure 3: **Correlation between results for Exp. 2 and Exp. S1**. Correlation between the fraction of participants who chose the expected program for the *"Choose the better program"* instruction, compared with the fraction of participants in Exp. S1 who chose the expected program when instructed to *"Choose the better robot"*.

that impacted their preferences. While it is unclear precisely why this is the case from a theoretical perspective, we consider this direction an exciting opportunity for future work to further explore the distinction in how people intuitively categorize concepts such as *"Agent"*, *"Program"* and *"Robot"*.

In our correlation analysis, we predicted that the trials people preferred across these two instructions would be correlated. As expected, we find significant correlations in the Generalization condition, suggesting that people are likely using similar Generalization metrics to evaluate agents across these two experiments. However, another unexpected finding is that we do not observe a significant correlation between which trials people prefer across the *Better Program* and *Better Robot* instructions (Figure 3) for the Action Efficiency and Representation Conditions. This might be explained by the same factors that led people to have different preferences in these two experiments for the Action Efficiency and Representation Efficiency conditions. In the Action Efficiency condition, people consistently choose the expected program in nearly all trials for the *Better Program* instruction, while there is much higher variance in people's preferences for the *Better Robot* condition. The converse is true for the Representation Efficiency condition: we observe higher variance in responses for the *Better Program* instruction, and lower variance for *Better Robot*. This pattern might help explain why there is minimal correlation, since one of the dimensions has very little variance to be explained by the other.

2 Comparing Programmers and Non-Programmers

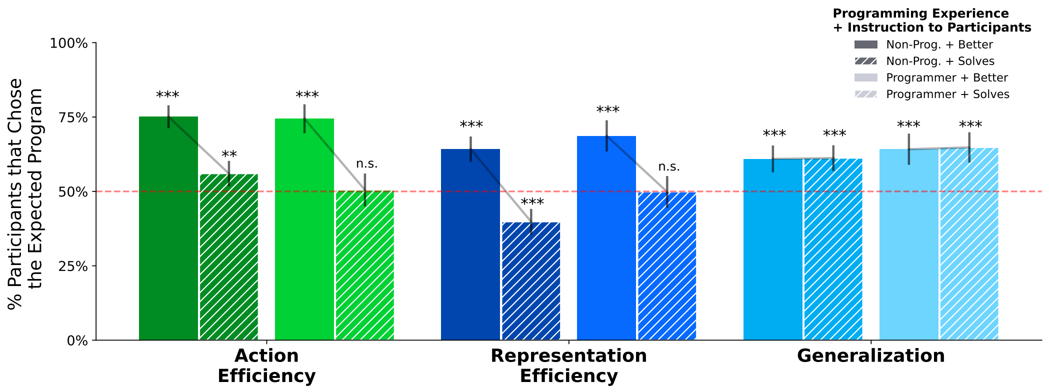

Figure 4: **Experiment 2 responses by condition, segmented by programming experience.** Similar to Figure 6 in the main text, this shows the fraction of participants who chose the expected program for each of three conditions (Action Efficiency, Representation Efficiency, Generalization), across two different instructions (*"Choose the better program"* or *"Choose the program that solves more mazes"*).

We found several differences between participants coded as programmers compared to non-programmers in the *Solves* condition for Experiment 2 (Figure 4 striped bars, and Table 5; Supplementary Materials). In the Action Efficiency and Representation Efficiency conditions for the *Solves* task, we found that programmers had no bias (i.e. $\mu \approx .5$), which suggests that experienced programmers in the both the *Better* and *Solves* groups understood both the experiment and the specific task they were being asked to perform. On the other hand, completely new programmers showed a bias to predict higher Generalization for programs which have longer code (Representation Efficiency $\mu < .5$) or fewer actions in the video shown (Action Efficiency $\mu > .5$).

**Condition**	**Programming**	**Task**	μ	p
Action Efficiency	Non-Programmers	Better	.75	$< .001$
Action Efficiency	Non-Programmers	Solves	.56	$< .01$
Action Efficiency	Programmers	Better	.74	$< .001$
Action Efficiency	Programmers	Solves	.50	.91
Representation Efficiency	Non-Programmers	Better	.64	$< .001$
Representation Efficiency	Non-Programmers	Solves	.40	$< .001$
Representation Efficiency	Programmers	Better	.69	$< .001$
Representation Efficiency	Programmers	Solves	.50	1.0
Generalization	Non-Programmers	Better	.61	$< .001$
Generalization	Non-Programmers	Solves	.61	$< .001$
Generalization	Programmers	Better	.64	$< .001$
Generalization	Programmers	Solves	.65	$< .001$

Figure 5: **Experiment 2 mean values and Binomial significance tests.** μ is the fraction of participants who chose the same program as we predict, and p values are for two-sided Binomial tests against a random baseline $P = .5$. Also see Figure 4 in Supplementary Materials.

When splitting our correlation analysis between programmers and non-programmers (Figure 6, Supplementary Materials), we find a significant correlation ($\rho = .57$, $p < .01$) for the Action Efficiency condition, for programmers only. This suggests that programmers are more consistent than non-programmers in assessing Generalization, and in using this metric to determine *'Which program is better?'*. This correlation being significant only in the Action Efficiency condition, in which both programs have equal Generalization according to our model, and not the Generalization condition, further suggests that programmers are estimating Generalization in a way that is different from our model.

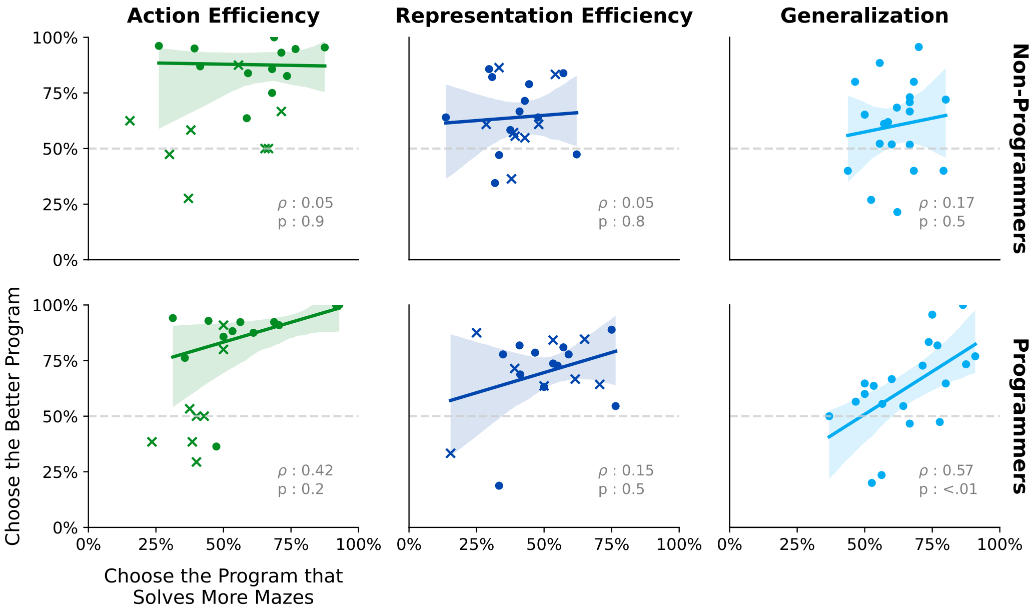

Figure 6: **Experiment 2 correlation between *Better* and *Solves* conditions, segmented by programming experience.** Similar to Figure 8 in main text. Correlations for each test trial in Experiment 2, between the fraction of participants who selected a given program with the prompt *Choose the better program* and the fraction who chose the same program with the prompt *Choose the program that solves more mazes*. Circles are trials where both programs succeeded at solving the mazes, X's are trials where both programs failed. We find a significant Spearman correlation ρ only for non-programmers in the Action Efficiency and Generalization conditions. Note that for the Action Efficiency condition, correlations omit failures (X's), whereas correlations include failures in the Representation Efficiency condition.

3 Analyzing Excluded Data

While we note that the proportion of excluded data (19%) in Experiment 2 was not out of keeping with many current empirical studies, it is useful and important to consider how our results change when including this data in the analysis (Figure 7).

Action Efficiency Failure Trials For our first analysis, we include the trials for Action Efficiency where both programs failed to reach the goal, which only affects the results for the Action Efficiency Condition. We excluded these trials from our main analysis since Action Efficiency only evaluates how fast a program *solves* a maze, but this does not account for how quickly a program *fails* a maze. When including these trials, we find the fraction of participants who chose the expected program drops for both instructions (*"Choose the better program"* and *"Choose the program that solves more mazes"*). For the *Solves* instruction, including Action Efficiency failure trials reduces the mean fraction of participants who chose the expected program (from $\mu = .88$ to $\mu = .75$), which does not affect the Binomial test results ($p < .001$). However, for *Solves* instruction, this changes the Binomial p-value from being significant ($\mu = .60$, $p < .001$) to non-significant ($\mu = .54$, $p = .16$).

Our results for this condition in the main text ($p < .001$) went against our prior expectation that participants given the *'Solves more mazes'* instruction would *not* choose the faster program at a greater than chance rate. Surprisingly, when Generalization was equal, participants in Exp. 2 predicted that programs which solved one maze faster would also be able to solve a greater number of mazes. This result may imply that when people fail to recognize a difference in Generalization, they fall back on features such as Action Efficiency and Representation Efficiency to estimate the number of mazes that a program will solve. Moreover, the change in results when including Action Efficiency failure trials for the *Solves* instruction potentially suggests that people may, in addition, reason that programs which take more steps to fail a maze will also solve more mazes. Such reasoning (if true) may be rational for domains such as ours, since many of the programs which have very high generalization (e.g. "wall follower" algorithms) are also slow to solve mazes. If this were the case, then this would also explain the decrease in means for the *Better* instruction, since people would estimate that slower-failing programs have higher Generalization and are thus "better"

Low Effort Participants For our second analysis, we include data for participants who were excluded due to low effort, either for having too short of an overall completion time, too little time per trial during the test phase, or failing our attention checks. In this analysis, we exclude data for failure trials in the Action Efficiency condition, as with our analysis in the main text. We find our analysis results are relatively unchanged when including this data, with means μ differing by at most .02, and $p - values$ remaining similar.

**Condition**	**Not Excluded**	**Task**	μ	p
Action Efficiency	-	Better	.88	$< .001$
Action Efficiency	Action Eff. Failures	Better	.75	$< .001$
Action Efficiency	Low Effort	Better	.85	$< .001$
Action Efficiency	-	Solves	.60	$< .001$
Action Efficiency	Action Eff. Failures	Solves	.54	.16
Action Efficiency	Low Effort	Solves	.60	$< .001$
Representation Efficiency	-	Better	.66	$< .001$
Representation Efficiency	Action Eff. Failures	Better	.66	$< .001$
Representation Efficiency	Low Effort	Better	.65	$< .001$
Representation Efficiency	-	Solves	.44	$< .005$
Representation Efficiency	Action Eff. Failures	Solves	.44	$< .005$
Representation Efficiency	Low Effort	Solves	.44	$< .005$
Generalization	-	Better	.62	$< .001$
Generalization	Action Eff. Failures	Better	.62	$< .001$
Generalization	Low Effort	Better	.60	$< .001$
Generalization	-	Solves	.63	$< .001$
Generalization	Action Eff. Failures	Solves	.63	$< .001$
Generalization	Low Effort	Solves	.61	$< .001$

Figure 7: **Experiment 2 results with excluded data.** Analysis results for Experiment 2 when including excluded data. For our main analysis, we excluded data based on two factors: first, we excluded low-effort participants who failed attention checks ('Low Effort'), and second, we excluded trials for the Action Efficiency condition where both programs failed instead of succeeding ('Action Eff. Failures'). For comparison, we also include results from the main analysis ('-'), where both are excluded.

4 Full Stimuli for Experiment 1

Here, we provide further examples of the stimuli we use in our experiments, elaborating on Figure 2 in the main text. We list all maze and program stimuli used in Experiment 1, organized by condition - Action Efficiency (Figure 8), Representation Efficiency (Figure 9), and Generalization (Figure 10). Each figure shows the maze, which program was preferred by our model (Program 1; ordering was randomized in our experiments), and the fraction of participants who chose the program predicted by our model.

We find that in 8/9 trials, people preferred the same program as expected according to our model. The last trial was in the Generalization condition (Figure 10). This result suggests that people are not estimating Generalization in the same way as our model, though there are multiple possible reasons why this would be true. For example, people might be estimating Generalization in the same way as our model, but are failing to comprehend certain programs; alternatively, people may be estimating Generalization in an entirely different way, for example approximation by sampling a small, biased selection of mazes which the program will solve or fail to solve.

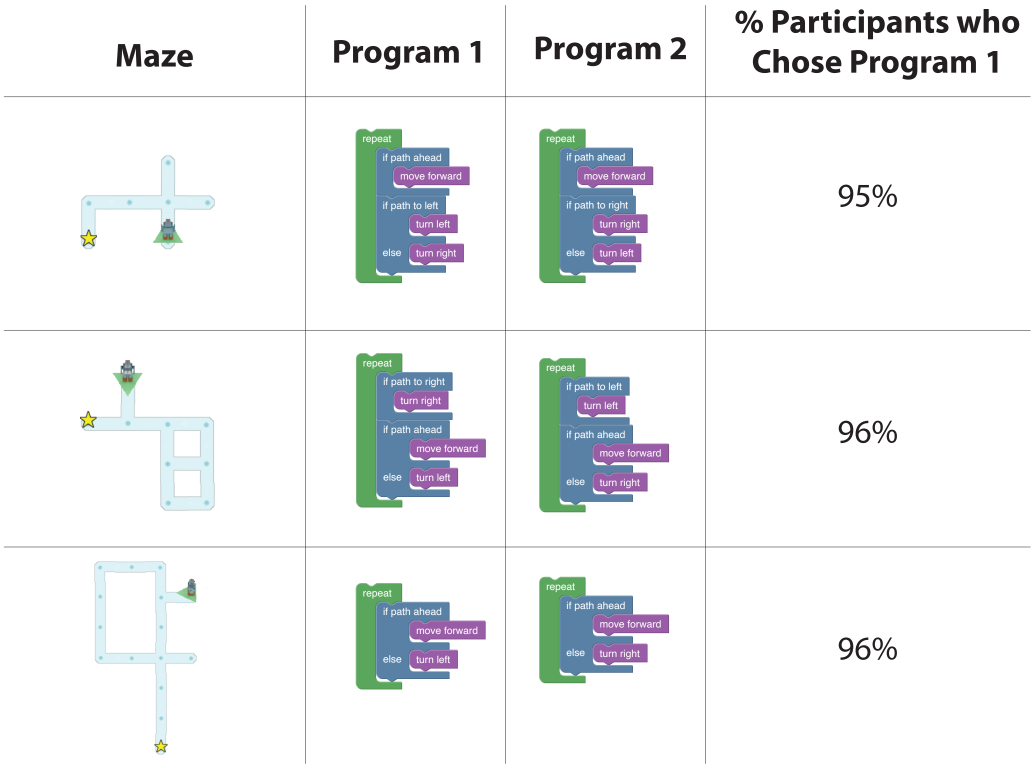

Figure 8: **Experiment 1 Action Efficiency Stimuli**. For each trial, Program 1 has better Action Efficiency (fewer actions to reach the goal) compared with Program 2 according to our model. Both programs have equal Representation Efficiency and Generalization. Note that in the experiment, program ordering is randomized for participants.

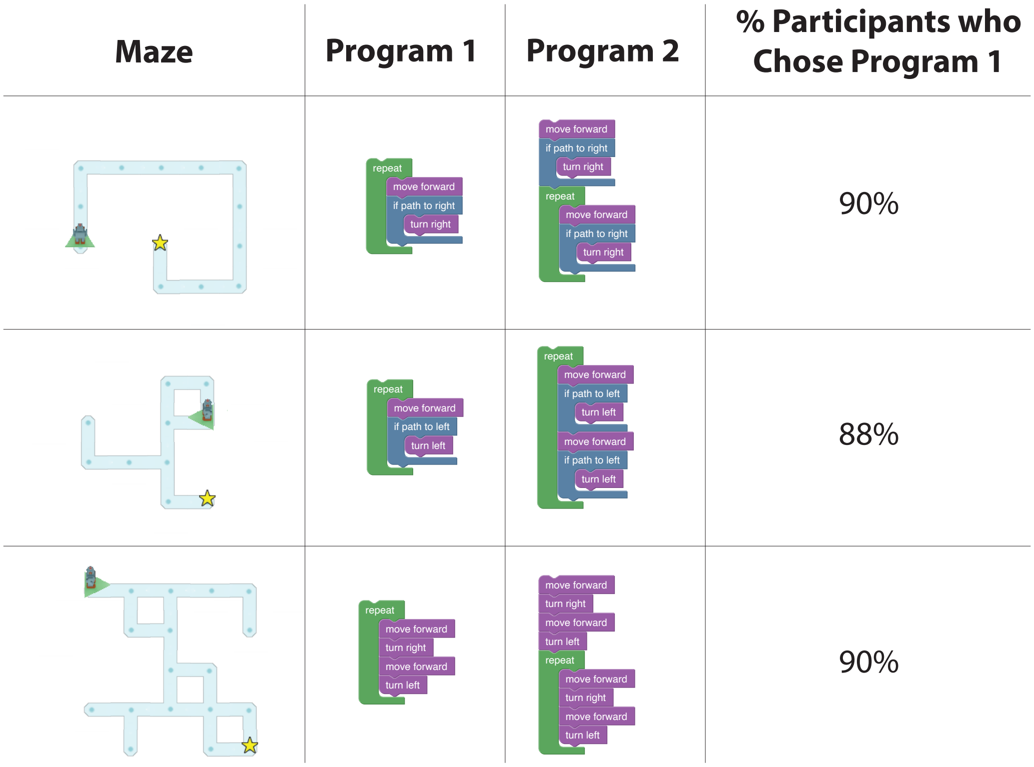

Figure 9: **Experiment 1 Representation Efficiency Stimuli**. For each trial, Program 1 has better Representation Efficiency (shorter code length) compared with Program 2 according to our model. Both programs have equal Action Efficiency and Generalization.

Maze	Program 1	Program 2	% Participants who Chose Program 1
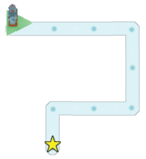	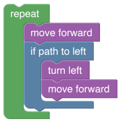	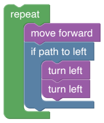	82%
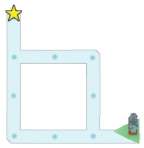	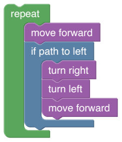	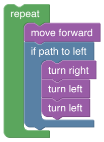	35%
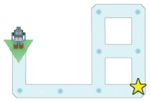	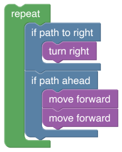	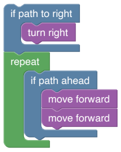	70%

Figure 10: **Experiment 1 Generalization Stimuli**. For each trial, Program 1 has higher Generalization (solves more mazes) compared with Program 2 according to our model. Both programs have equal Action Efficiency and Representation Efficiency. In this condition, we find that people prefer Program 1 for 2/3 trials (Top and Bottom), but people instead show a preference for Program 2 in one trial (Middle).
